# Xanthogranulomatous cholecystitis: diagnostic complexity and review of the literature

**DOI:** 10.1093/jscr/rjad308

**Published:** 2023-06-17

**Authors:** William Arnott, Jemima Hutchins, Tanishk Malhotra, Yathurshika Ketheesan, Lilly Steinberg, Lucy Carter, Jason Diab, King Wong

**Affiliations:** Department of General Surgery, The Tweed Hospital, Tweed Heads, NSW, Australia; John Flynn Private Hospital, Tugun, QLD, Australia; School of Medicine, Bond University, Robina, QLD, Australia; School of Medicine, Griffith University, Southport, QLD, Australia; Department of General Surgery, The Tweed Hospital, Tweed Heads, NSW, Australia; Department of General Surgery, The Tweed Hospital, Tweed Heads, NSW, Australia; School of Medicine, Bond University, Robina, QLD, Australia; Department of General Surgery, The Tweed Hospital, Tweed Heads, NSW, Australia; School of Medicine, Bond University, Robina, QLD, Australia; Department of General Surgery, The Tweed Hospital, Tweed Heads, NSW, Australia; School of Medicine, Bond University, Robina, QLD, Australia; Department of General Surgery, The Tweed Hospital, Tweed Heads, NSW, Australia; School of Medicine, Bond University, Robina, QLD, Australia; Department of General Surgery, The Tweed Hospital, Tweed Heads, NSW, Australia; John Flynn Private Hospital, Tugun, QLD, Australia; School of Medicine, Bond University, Robina, QLD, Australia; School of Medicine, Griffith University, Southport, QLD, Australia; Department of General Surgery, The Tweed Hospital, Tweed Heads, NSW, Australia; John Flynn Private Hospital, Tugun, QLD, Australia; School of Medicine, Bond University, Robina, QLD, Australia; School of Medicine, Griffith University, Southport, QLD, Australia

## Abstract

We report the case of a 39-year-old male presenting with acute onset vomiting and diarrhoea. Initially treated empirically for gastroenteritis, imaging later confirmed a complicated episode of cholecystitis with fistular formation and intra-abdominal cyst. Following cholecystectomy, histology confirmed a case of xanthogranulomatous cholecystitis (XGC). This paper presents a detailed summary of the condition alongside a literature review of all available episodes of XGC since 2017 with the aim of highlighting diagnostic conclusions regarding the nature of the disease and its clinical manifestations.

## INTRODUCTION

Xanthogranulomatous cholecystitis (XGC) is a rare benign inflammatory condition secondary to the extravasation of bile into the intramural tissue of the gallbladder. This generates reactive inflammatory changes with progressive proliferative fibrosis of the gallbladder wall [1]. Radiological assessment displays some pathognomonic features such as diffuse gallbladder wall thickening and intramural nodules, yet histological analysis provides the definitive diagnosis post cholecystectomy [2]. We herein present a case of XGC including a review of the literature.

## CASE REPORT

A 39-year-old Caucasian male presented to their general practitioner with worsening abdominal pain in the context of a recent admission to hospital for severe vomiting and diarrhoea. The patient had no significant medical history, was a non-smoker and did not take any regular medications. He had undergone a laparoscopic gastric bypass 6 months prior without complication.

On presentation, he was haemodynamically stable with tenderness to palpation maximally in the right upper quadrant. Ten days prior his biochemical markers showed a raised white cell count (19.9 × 10^9^/L), elevated C-reactive protein (557), an acute kidney injury (creatine of 376 umol/L), and deranged liver function with an elevated bilirubin of 32 umol/L, and raised GGT 262 u/L and ALP 193 u/L.

**Figure 1:**
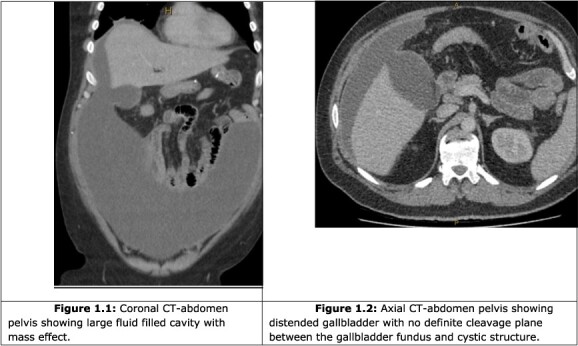
CT abdomen and pelvis.

**Figure 2:**
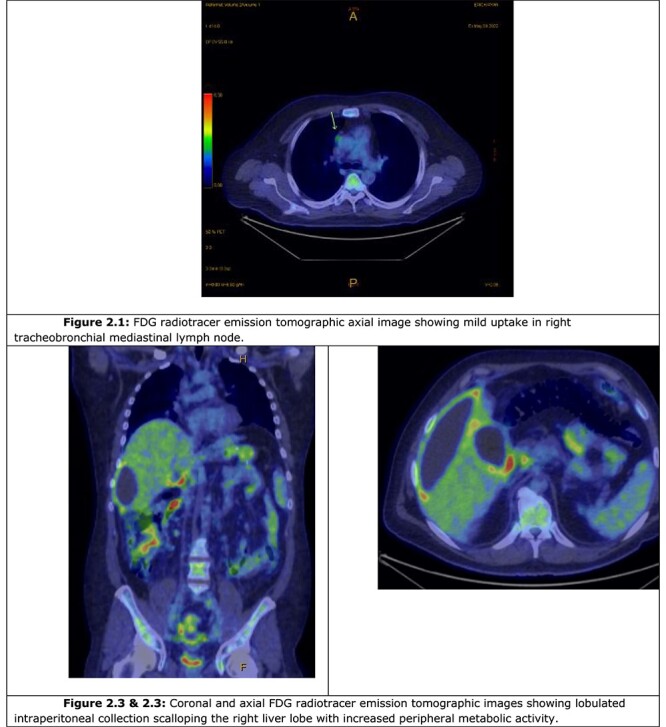
PET study.

A computer tomography (CT) abdomen and pelvis with contrast was ordered (Fig. 1), which demonstrated a fluid-filled abdominal collection continuous with a distended gallbladder. The patient was referred to a local general surgeon, and underwent a positron emission tomography (PET) study to rule out intra-abdominal malignancy (Fig. 2). The differentials included primary mucinous tumour of the gallbladder, duplication cyst and pseudomyxoma peritonei.

**Figure 3:**
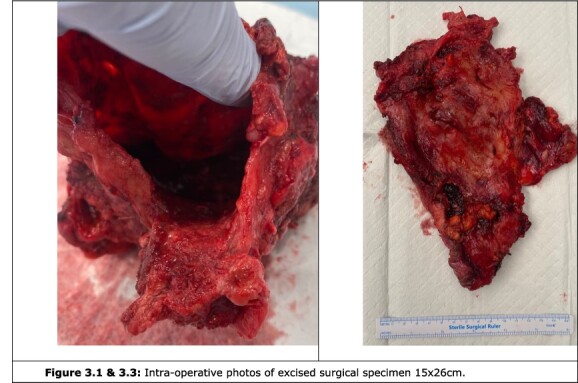
Intra-operative photos of excised cyst.

Following multi-disciplinary team discussion, the decision was made to undergo an elective laparotomy and debridement of the presumed inflammatory cyst plus cholecystectomy and intra-operative cholangiogram. Intraoperatively, a large intraperitoneal cyst extending from the supra-hepatic space and adherent to the pelvis, bladder and retroperitoneum was excised (Fig. 3). The cyst contained purulent material with a thick rind. On further evaluation, there was an evidence of gallbladder perforation into the cyst with associated necrosis.

Histopathology revealed a large irregular walled cystic structure with haemorrhagic exudate as well as a fibrotic cholecystectomy specimen. There was an evidence of mixed inflammation consisting of lymphocytes, neutrophils, foamy histocytes and occasional giant cells consistent with xanthogranulomatous inflammation and cholecystitis. He remained on the ward for 3 days with no post-operative complications. At 6-week follow-up, he was asymptomatic.

## DISCUSSION

XGC is characterized by the accumulation of lipid-laden macrophages, termed xanthoma cells, in the intramural lining of the gallbladder wall secondary to the inflammatory response to extravasated bile [3, 4]. The disease remains rare, with an estimated incidence of only 1.46% of all cholecystectomy specimens evaluated from a sample of over 12 000, with an observed prevalence of 2:1 males to females [4].

The mechanism of bile extravasation remains unclear, but may be related to either mucosal ulceration from gallstones [5], or the rupture of Rokitsanky–Aschoff sinuses (Luschka crypts)—which are deep mucosal outpouchings of the gallbladder that extend into or though the muscular layers of the wall [6, 7].

The accumulation of bile within the peri-muscular layers of the gallbladder activates macrophages that phagocytose the lipid molecules and bacteria within the bile and form foamy macrophages. These lipid burdened macrophages activate fibroblasts through chemotactic and mitogenic stimulation, and encourage the production of collagen fibres [8, 9]. There is resultant hyaline degeneration, formation of intramural nodules and thickening of the gallbladder wall [3, 4, 10]. The resulting erosion may lead to perforation of the gallbladder, peritoneal or hepatic abscess formation, and gastric or bowel fistulae [11].

The research team identified a total of 629 reported cases of XGC (Tables 1 and Table 2). The mean age of these episodes was 61, with slightly more prevalence in males than females, with a total of 273 females to 341 males found in studies reporting gender, reflecting a ratio of F:M of 1:1.25.

**Table 1:** A literature review of case reports on XGC

Author (year)	Patient presentation	Blood and biochemical	Imaging and diagnostic procedures	Management	Diagnostic histopathology	Outcome
Gupta (2021)	A 71-year-old female presenting with 3-month history of abdominal pain and fever.	*None provided*	**Abdominal US:** thickening of gallbladder wall. **Whole-Body 18F-FDG PET-CT**: intensely FDG avid circumferential nodular mural thickening in the gallbladder fundus.	Due to high suspicion of malignancy from radiological findings, Cholecystectomy was performed *(details not provided).*	Gallbladder mucosa was ulcerated with flattened mucosa. Gallbladder wall had dense mixed inflammatory infiltrate rich in histiocytes, infiltrating muscle. Consistent with a diagnosis of XGC.	*None provided*
Morare (2020)	A 57-year-old male presenting with 2-week history of RUQ pain, weight loss, anorexia and night sweats. Hemodynamically stable, apyrexial, mild Pallor, nil Jaundice, 5 cm hepatomegaly.	**GGT 318 U/l** (<68 U/l) **ALP 332 U/l** (53–128 U/l) **ALT 58 U/l** (10–40 U/l) **Total Bilirubin 38 μmol/l** (5–21 μmol/l) **Conjugated Bilirubin 22 μmol/l** (0–3 μmol/l) **WCC 13.37** (3.92–10.40 × 109/l) **CRP 160 mg/l** (<10 mg/l)	**Abdominal CT:** enlarged thickened gallbladder with multiple stones, two liver abscesses, fistulous tract to the hepatic flexure with abscess. **Colonoscopy:** no tumour or fistula visualized. **Abscess Drain:** nil organisms, amoebic or hydatid disease.	Laparotomy: adhesiolysis, cholecystectomy, segmental resection of colon and fistula with primary anastomosis. Findings: thickened gallbladder with significant adhesions, and a cholecystocolonic fistula.	**Histopathology:** 80 × 50 × 15 mm gallbladder with ulcerated mucosal surface and irregular fibrino-purulent exudate. Fat necrosis and foamy histiocytes. Fistula 20 mm made up of bowel mucosa.	Discharged day 4. No follow-up recorded
Rahman (2020)	A 56-year-old male smoker with RUQ pain, vomiting, weight loss and fever. Examination found a firm, grossly distended abdomen, with no jaundice.	Elevated **Total Bilirubin**. **Leukocytosis**. **CA19-9** and **CEA** normal. (*Values not provided)*	**Abdominal US:** gallbladder wall thickening with multiple intramural foci suggestive of adenomyomatosis. **Abdominal CT:** inflammatory changes with gallbladder thickening and pericholecystic fat stranding. **ERCP**: nil dilatation. Nil filling defect of common bile ducts.	After radiological findings suspected acute cholecystitis with possible perforation, a cholecystectomy was performed (further details not provided).	**Histopathology:** Serosal adhesion with thickened, ulcerated, fibrotic gallbladder wall; an abundance of histiocytes and giant cell reaction; acute and Chronic inflammation with bile dissipation, rendering a diagnosis of XGC.	*None provided*
Zakaria (2021)	A 66-year-old male with 2 months of worsening RUQ pain.	**Hyperleukocytosis** at 17 000/uL (4000–10 000), Elevated **CRP** at 215 mg/L (<5 mg/L), and a **biological cholestasis** (further *values not provided)*	**Abdominal US:** diffusely thickened gallbladder wall. Multiple hypoechoic nodules. No duct dilatation. **Abdominal CT:** thickened wall at the site of hypodense nodules. **Abdominal MRI:** area of mucosal defect w/ intramural collection and an adjacent liver collection.	After radiological findings suspected XGC, cholecystectomy with drainage of the hepatic abscess was performed subcostally.	Histopathology of the specimen showed pseudotumoral features. The presence of foamy histocytes, inflammatory infiltrate and parietal fibrosis confirmed the diagnosis of chronic XGC.	*None provided*
Alcazar (2020)	A 73-year-old female presented with RUQ pain and vomiting.	Blood tests indicated dissociated cholestasis and an increase in acute phase reactants *(Values not provided)*	**Abdominal US:** hydropic gallbladder with multiple gallstones and mucosa with a reticular appearance. **Abdominal MRI:** showed a hydropic gallbladder with gallstones, thickened walls and multiple thick internal septum.	Based on radiological suspicion of either gallbladder carcinoma or XGC laparoscopic cholecystectomy was performed. This revealed acute cholecystitis with a contained perforation.	Histopathology indicated sparkling histiocytes, multinucleated giant cells, pigmented particles and chronic inflammatory infiltrate. These findings confirmed the suspected diagnosis of XGC.	*None provided*
Neychev (2018)	A 67-year-old female presented with RUQ pain, nausea and vomiting for 1 month, with 10 kg weight loss. Examination found a palpable, poorly defined, tender mass in RUQ and tachycardia.	**WBC** 10.2 × 10^9/L **Hb** 135 g/L **ASAT** 18 U/L **Total bilirubin** 6.8 mmol/L **Direct bilirubin** 3.6 mmol/L **CA 19-9** 14.4 U/mL **CEA** was 0.6 ng/mL	**Abdominal US/CT:** large, ill defined, heterogeneous mass completely replacing the gallbladder with extensive involvement of adjacent liver segments, duodenum, head of pancreas and hepatic flexure of colon. Several enlarged loco-regional lymph nodes. **MRCP:** nil intra/extrahepatic biliary tree dilatation.	Exploratory laparotomy revealed a mass occupying the subhepatic space with adhesions to omentum, adjacent liver segments, hepatoduodenal ligament, second portion of duodenum, proximal one-third of transverse colon. Resection of the mass, adjacent liver segments and loco-regional lymph nodes was performed.	Histopathology - diffuse thickening of gallbladder wall (up to 1.5 cm). Consistent with diffuse XGC. Deep ruptured Rokitansky–Aschoff sinuses penetrating the muscle layer. Multiple Foci of foamy macrophages and xanthoma cells.	Patient discharged 7 days post operation. At 6-month follow-up patient remains asymptomatic and healthy.
Aslam (2020)	A 40-year-old female presents with gradually increasing RUQ pain for 3 months in the context of medically managed acute cholecystitis 8 months earlier and previous excision of hydatid cyst.	Blood Chemistry Unremarkable *No Details Provided*	**Abdominal US:** inflamed gallbladder with cholelithiasis and a dilated common bile duct.	Laparoscopic cholecystectomy converted to open cholecystectomy due to gastric, duodenal and omental adhesions. Resection included the gallbladder, local lymph nodes and part of liver segment 4. Empyema drained.	Resected gallbladder showing dense acute and chronic inflammatory changes with foamy histiocytes and giant cells confirming a diagnosis of XGC.	Discharged 5 days post-operatively.
Alammari (2022)	A 70-year-old male presenting with 2 months of sharp RUQ pain radiating to the right shoulder, nausea. Medical history of hypertension, Stable Angina, T2DM.	**Haemoglobin** 14.5 g/dL **White Blood Cell** 14 000/mL **CRP** 18.2 mg/dL **Platelet** 390 000/mL **ESR** 52 mm/h **Total Bilirubin** 1.4 **Albumin** 3.1 g/dL **ALP** 110 U/L **GGT** 81 U/L 15–85 **ALT** 60 U/L 14–63 **AST** 41 U/L 15–37	**Abdominal CT**: diffuse thickening of gallbladder wall with collapsed lumen, suspicious for malignancy. Nil presence of abscess, fistula, hepatic infiltration**.**	Open cholecystectomy showed significantly enlarged gallbladder.	Histopathology showed lipid-laden macrophages along with chronic inflammation consistent with XGC	Oral feeding started day 2 post op. At one month follow up patient was asymptomatic
Ramia (2020)	A 38-year-old female with an incidental finding of a hepatic hilum lesion on PET/CT for metastatic melanoma surveillance.	Blood Lab Values - Normal *(details not provided)*	**PET/CT**: hypermetabolic lesion in the hepatic hilum causing dilation of gallbladder. **MRI:** polypoid mass in gallbladder, concerning for metastasis.	Cholecystectomy with 1.5 cm resection of liver parenchyma for clear margins found a dilated GB with thickened wall and inflammation of the hilar plate.	Histopathology demonstrated Lymphoplasmacytic inflammatory infiltrate in GB wall. Abundant histiocytes with brown pigment in cytoplasm and formed nodules. Erosion and Ulceration of mucosa.	*None provided*
Khan (2019)	A 60-year-old male with 1 month of RUQ pain with previous choledoco-cholelithiasis managed with ERCP and stenting.	**CA 19-9** 22.9 U/ml. Other Labs within range (details not provided)	**CT:** infiltrative mass in GB Fundus involving right lobe of liver and hepatic flexure of colon.	Radical cholecystectomy, extended right hepatectomy and right hemicolectomy with ileotransverse anastomosis. Intraoperative findings showed the GB infiltrating the right hepatic lobe and hepatic flexure.	Final histopathology confirm a diagnosis of XGC as the cause of the mass.	Post-operative intra-abdominal collection and AKI managed with drainage and haemodialysis
Lee (2020)	A 64-year-old female referred by GP with an ultrasound finding of a 2.3 cm GB Lesion on a background of cholelithiasis and elevated ferritin.	Normal **CA 19-9** at 12 u/ml	**US, CT, MRI**: ill-defined gallbladder interface with liver and abutment to colon and duodenum. Enlarged upper abdominal and periportal lymph nodes measuring 11 mm.	Diagnostic laparoscopy showed multiple 1–2 cm lesions in LUQ of peritoneum with duodenum and colonic involvement. Proceeded with open cholecystectomy, partial hepatectomy, wedge excision of the colon, partial duodenectomy, Portal Lymphadenectomy.	Cystic margin showed reactive atypia with nil dysplasia. Pathology of gallbladder confirmed XGC showing lymphohistiocytic inflammatory infiltrate, multifocal abscesses, multiple multinucleated giant cells (Touton Type Giant Cells). Nil Dysplasia or Carcinoma.	Superficial surgical site cellulitis.
Garg (2018)	A 62-year-old female presents with RUQ pain, vomiting and tachycardia.	**Hb** 9.1 g/dL **WCC** 17 × 10^9/L **K+** 2.8 mEq/L All other bloods in normal range (details not provided)	**Abdominal US:** collapsed GB with a smooth curvilinear mass and posterior acoustic shadowing at distal duodenum. **Abdominal CT:** mass-like thickening of GB wall with multiple hypoattenuating nodules. **Gastroscopy:** Pyloric obstruction	Partial cholecystectomy and primary repair of fistula over a T-tube, with a retrocolic gastrojejunostomy. Intraoperative findings showed a GB mass adherent to duodenum and colon, with narrowing at gastric outlet. GB fistula to second part of duodenum.	Histopathology confirmed diagnosis of XGC with inflammatory infiltrate composed predominantly of foamy macrophages, mixed with lymphocytes, plasma cells and few neutrophils	*None provided*
Nacif (2017)	A 42-year-old female with 6 kg weight loss presented with epigastric pain and jaundice.	**Serum Bilirubin** 9.3 mg/dL (0.2–1.2 mg/dL) All other bloods in range	**CT/MRCP:** gallstones present, with asymmetrical GB wall thickening, as well as a contiguous hepatic hilar mass infiltrating segment 4b, common bile duct and bilateral hepatic ducts with associated intrahepatic biliary dilatation. Nil lymphadenopathy.	A right trisectionectomy with cholecystectomy was performed with a complete extirpation of the extrahepatic bile duct, hilar lymphadenectomy and double Roux-en-Y hepaticojejunostomy.	**Histopathology**: chronic inflammation with xanthogranulomatous changes and no malignancy.	Discharged Day 13 post op. 10-year follow up patient is asymptomatic
Nacif (2017)	A 66-year-old male presenting with epigastric and RUQ pain, fever and jaundice.	Serum Bilirubin 3 mg/dL (status post biliary drainage) **WBC** 18.5 × 10^9/L *(further detail not provided)*	**CT/MRCP**: collapsed gallbladder with gallstones and focal malignant appearing wall thickening. Contiguous infiltration of hepatic segment 4b and biliary confluence, with associated intrahepatic biliary dilatation and a 1 cm spiculated hilar lymph node.	Exploratory laparoscopy converted to open right trisectionectomy with cholecystectomy and complete extirpation of the extrahepatic bile duct, hilar lymphadenectomy and double Roux-en-Y hepaticojejunostomy.	Perioperative frozen-section analysis of the suspicious hilar lymph node was negative for malignancy. Histopathology later confirmed the diagnosis of XGC (details not provided).	Mild hepatic insufficiency (grade 1 hepatic encephalopathy and Serum Bilirubin 8.5 mg/dL) 9-year follow up patient asymptomatic
Nacif (2017)	A 65-year-old male presented with jaundice, choluria and anorexia. Medical history of hypertension, glucose intolerance, heavy smoker and prior left nephrectomy	Serum Bilirubin 6.8 mg/dL *(further detail not provided)*	**Abdominal CT**: gallstones with asymmetric gallbladder wall thickening, contiguous hilar mass infiltrating bilateral hepatic ducts and contacting the right hepatic artery and portal vein, without any apparent plane of separation.	En bloc resection of the gallbladder, hepatic segments IVb and V, extrahepatic bile duct, hilar lymphadenectomy and Roux-en-Y hepaticojejunostomy	Intraoperative frozen section of lymph node - negative for malignancy Histopathology confirms chronic cholecystitis with focal areas of XGC. *(further details not provided)*	Discharged on day 9 post op Patient well at 7-year follow up
Fafaj (2018)	A 64-year-old male presented with RUQ pain, nausea and diarrhoea. Previous medical admissions for multiple intrahepatic abscesses, sepsis, diabetic ketoacidosis in the context of Type 2 DM, Chronic renal failure, Chronic Hepatitis C, Coronary Artery Disease	**WBC** of 3.84 k/uL **Hb** 9.4 g/dL **AST** 56 U/L **ALT** 63 U/L **ALP** 261 U/L **Total bilirubin** 1.0 mg/dL **CA 19-19** 53 U/mL (<36 U/mL) **CEA** at 1.7 ng/mL (<2.9 ng/mL).	**Abdominal US**: hypodense areas in lateral and medial segments of left lobe. Left Portal Vein Thrombus. **Abdominal CT**: extensive thrombosis of portal system. **ERCP**: Choledocholithiasis left biliary tract occlusion secondary to mass. Biopsy showed benign fragments of fibrous tissue with bile crystalline.	**ERCP**: biliary stent placement. **Diagnostic Laparoscopy**: omental adhesions with significant inflammation of segments 4 and 5 of liver with gallbladder diffusely thickened. Hard mass in segment 4. **Laparotomy**: cholecystectomy with an extended left hepatectomy.	Washings negative for malignancy. Histopathology showed transmural inflammatory process in the gallbladder with mucosal ulceration and the mass lesion composed of sheets of foamy histiocytes admixed with plasma cells, lymphocytes, collagen fibres and scattered giant cell, confirming diagnosis of XGC.	Discharged on day 8 post operatively into extended care facility
Reghunath (2020)	A 74-year-old female presented with a 6-month history of RUQ pain. Negative Murphy's Sign on physical examination.	LFT in normal limits (no other bloods provided)	**Abdominal USS:** thickened gallbladder wall with echogenic sludge and intramural hypoechoic nodules. **Abdominal Contrast CT:** homogenous enhancement of thickened gallbladder wall with multiple intramural cystic areas. Focal breach in GB near liver with fat planes maintained. **Abdominal MRI**: T2 Intramural hyperintense nodules in GB wall. **FNAC:** Taken from fundus of gallbladder. Showed polymorphs and foamy histiocytes	Based on FNAC and inflammatory image from radiology, this patient underwent open simple cholecystectomy.	No postoperative histology provided	*None provided*
Haring (2021)	An 84-year-old male presents with icterus on a background of conservative treatment for acute cholecystitis.	**CA19-9** 4240 kU/l	**Imaging:** malignant changes. **ERCP**: obstructive choledocholithiasis	Laparoscopic cholecystectomy	XGC	CA19-9 Levels decreased after surgery and patient recovered swiftly
Park (2020)	A 76-year-old male presents with postprandial abdominal pain and a palpable RUQ mass, in the context of a 2-month history of unexplained weight loss.	**CRP** 41.2 mg/L. **ALP** 19 6196 IU/L (<129) **GGT** 152 U/L (<69) **CA 19-9** 27.7 U/mL	**Abdominal CT:** asymmetrical thickening of GB wall with extensive liver involvement and multiple intramural hypoattenuated nodules. **Abdominal MRI:** gallstones, asymmetric wall thickening and a contiguous hepatic mass in segment V with right intrahepatic duct dilatation.	Laparoscopic cholecystectomy	Thickened GB. Serosa with dense adhesions. Ulcerated Mucosa. Xanthogranulomatous foci made up of lipid laden macrophages, fibroblasts and inflammatory cells	None provided
Shetty (2017)	A 52-year-old male presenting with a 2-day history of pain in the right hypochondrium and vomiting with a positive Murphy’s sign. Previous history of calculus cholecystitis.	**Hb** 11.4 g% **Total Leukocyte** - 15 800 cells/cumm ▪ Neutrophils 88% ▪ Lymphocytes - 10% ▪ Eosinophils - 2% **Blood Glucose** - 120gm/dl **Total Bilirubin** - 3.2 g/dl **Direct Bilirubin** - 2.0 g/dl **ALP** - 353 IU/L **AST** - 86 IU/L **ALT** - 57 IU/L	**Abdominal US:** multiple calculi (largest - 2 × 1 cm) With diffuse gallbladder wall thickening (9 mm)	Laparoscopic cholecystectomy was abandoned due to dense adhesions. Converted to an open cholecystectomy using a retrograde technique.	Extensive surface ulceration of GB with diffuse muscular wall infiltration by foamy histiocytes, lymphoplasmacytic infiltrate, cholesterol clefts and fibrosis. These findings suggested Lusckha ducts alongside the diagnosis of XGC.	There were no postoperative complications, and the patient was discharged. On their 2-month review, the patient was asymptomatic.
Alhomoud (2017)	A 59-year-old male presenting with a 2 day history of abdominal pain and jaundice with a medical history of chronic calculous cholecystitis.	*None provided*	**Abdominal US and CT Abdomen:** distended GB with concentric lobulated wall thickness (1.1 cm) with mud. Dilated CBD and intrahepatic biliary radicles, raising the possibility of cholangiocarcinoma cholecystitis with fluid collection.	ERCP with papillotomy and CBD. Subsequent laparoscopic cholecystectomy converted to open due to dense fibrous adhesions to hepatic parenchyma and transverse colon.	Examination of the specimen identified collections of foamy histiocytes with abundant lipid in the cytoplasm and admixed lymphoid cells, suggestive of XGC.	The patient was discharged on day 10 with no complications.
Zhang (2022)	A 42-year-old male with epigastric discomfort referred to hospital with concerns for possible gallbladder carcinoma detected on abdominal ultrasonography.	*None provided*	**MRI**: uneven thickening of the gallbladder wall with abnormal signals in adjacent liver parenchyma. Intramural nodules. **MIP Image and Ga-FAPI-04 PET Transversal Image:** intense Ga-FAPI-04 uptake in the gallbladder Intramural nodules showed uniform signal loss on the out-of-phase image which suggested fatty components.	Following the radiological findings, a cholecystectomy was performed (no further details provided).	Examination of the specimen revealed hyperplasia of fibrous tissue with inflammatory cell infiltration and formation of foam cells; features representative of XGC.	*None provided*
Wang (2022)	A 67-year-old male presenting with a painless mass in the right upper quadrant for 1 month.	**Ca19.9** - 5660.39 U/mL **Carbohydrate antigen 50** - >500.IU/mL **Carbohydrate antigen 24-2** - 98.28 IU/mL **CEA** - 5.99 ng/mL	**Abdominal CT:** thickened gallbladder and stones in the neck. **MRI:** enlarged gallbladder with irregularly thickened wall. Continuous mucosal line with hypoattenuated intramural nodules. **F-FDG PET/CT Fusion Image:** increased uptake to intramural nodules	Following the radiological findings, a laparoscopic cholecystectomy was performed.	granuloma with neutrophil infiltration and giant cells in the resected gallbladder wall alongside an accumulation of foamy histiocytes; features suggestive of XGC.	The patient recovered quickly and was discharged. Ca19.9 was monitored post operatively with a decrease in levels occurring.
Arya (2022)	A 64-year-old female presenting with recurrent, intermittent right upper abdominal pain for the past year. She had previously undergone a CABG with AVR.	*None provided*	**Abdominal USS:** features suggestive of cholecystitis with cholelithiasis. **Contrast enhanced CT:** asymmetric mural thickening in body and fundus of the gallbladder, with fistulous communication to the duodenum with intramural air and mild pneumobilia. **FNA of Gallbladder Lesion:** neutrophils, macrophages and plasma cells adherent to blood vessels lying in dispersed fashion. Foam cells, mesothelium like cells and multinucleated giant cells present.	Following the FNA, a radical cholecystectomy was performed. Intraoperative Findings: Duodenum adhered to the gallbladder Cholecysto-enteric fistula present Multiple omental adhesions at the gallbladder	Histopathology showed plasma cells, multinucleated giant cells, neutrophils, pigment, sheets of foamy macrophages and lymphoid aggregate present, suggestive of XGC. A microfocus (0.2 cm) of a poorly differentiated carcinoma, dysplastic epithelium and an intra-cystic papillary neoplasm suggests a malignant neoplasm accompanying the XGC.	*None provided*
Yang (2022)	A 59-year-old female presenting with recurrent RUQ pain and weight loss for 4 months.	**LFT** - Normal **UEC** - Normal **Ca19.9** - Elevated (63.98 KU/L; norm <35)	**Abdominal CT:** focal thickening of gallbladder wall with liver involvement of segment V and multiple hypodense nodules adjacent to the gallbladder	An exploratory laparotomy was performed with a cholecystectomy and partial liver resection. Findings were an atrophied, firm gallbladder with thickened fundus	Infiltration of foamy histiocytes, fibroblasts and inflammatory cells; supporting a diagnosis of XGC.	*None Provided*

**Table 2:** A literature review of case series on XGC

Author (year)	Cohort	Presentation	Blood and biochemical	Imaging and diagnostic procedures	Management	Diagnostic histopathology	Morbidity and mortality
Azari (2021)	Sample size: 27 ▪ M 17 ▪ F 10 Mean age: 64	**RUQ pain** 18 (67%) **Anorexia** 7 (26%) **Fever** 6 (22%) **Weight loss** 6 (22%) **Jaundice** 1 (4%)	Median Values Total Bilirubin (mg/dL) = 0.60 (0.43–1.32) White Cell Count (per microliter) = 12.3 (5.80–16.25) Serum creatinine (mg/dL) = 0.87 (0.66–1.02) INR = 1.15 (1.05–1.40) Platelet count (platelets/mcL) = 249 (197.50–297.50)	**US** ▪ Gallstones - 14/17 ▪ Gallbladder thickening >5 mm - 8/17 ▪ Pericholecystic fluid - 5/17 ▪ Intramural nodules - 1/17 **CT and MRI** ▪ Gallstones (CT): 9/21 ▪ Gallstones (MRI): 7/8 ▪ Pericholecystic fluid - 10/23 ▪ Gallbladder wall enhancement - 7/23 ▪ Gallbladder wall thickening >5 mm - 15/23 ▪ Intramural nodules - 2/23	▪ Laparoscopic cholecystectomy - 10/27 ▪ Open radical cholecystectomy - 3/27 ▪ Open simple cholecystectomy - 9/27 ▪ Open subtotal cholecystectomy - 5/27	▪ Mucosal ulceration - 18/27 (66%) ▪ Cytologic atypia - 7/27 (26%) ▪ Cholethiasis 22/27 (82%) ▪ Lymphohistiocytic infiltration - 24/27 (89%) ▪ Round foamy macrophages which are CD68 positive - 27/27 (100%) ▪ Findings suggestive of acute cholecystitis - 17/27 (63%) ▪ Findings suggestive of chronic cholecystitis - 22/27 (82%)	Mortality - 0 Morbidity ▪ Bile duct injuries repaired with Roux-en-Y hepaticojejunnostomy - 2/27 ▪ Acute kidney injury requiring temporary haemodialysis 1 ▪ Bleeding requiring transfusion 1 ▪ Postoperative endoscopic retrograde cholangiopancreatography - 2/27 ▪ Operative re-explorations for missed injuries (bile duct and duodenal) - 2/27 ▪ Drain placements for intra-abdominal collections - 2/27 ▪ 30-day readmissions for pain and surgical site infection requiring IV antibiotics - 2/27
Gunes (2021)	Sample size: 70 ▪ M 38 ▪ F 32 Mean age: 53.75	Features of chronic cholecystitis - 32 (45.7%) Features of acute cholecystitis - 23 (32.9%) Features of obstructive jaundice - 9 (12.9%) Features of acute biliary pancreatitis - 6 (8.5%)	*None provided*	**USS or MRI** ▪ Gallstones - 66 (94.2%) ▪ Wall thickness (>3 mm) - 41 (58.5%) ▪ Pericholecystic fluid - 4 (5.7%) ▪ Porcelain GB - 2 (2.85%)	▪ ERCP completed – 14/70 (20%) ▪ Laparoscopic cholecystectomy - 52/70 ▪ Open cholecystectomy - 18/70	Histopathological diagnosis of XGC from the gallbladder specimens. Findings not provided in the study.	N/A Mortality N/A Morbidity
Feng (2020)	Sample size: 100 ▪ M 55 ▪ F 45 Mean age: 55.6	**RUQ pain** - 97 **Jaundice** - 32 **Anorexia** - 25 **Abdominal distension** - 37 **Pyrexia** - 27 **Nausea and Vomiting** - 15 **RUQ mass** - 6	Elevated WBC - 30% Elevated blood bilirubin level - 32% Elevated Ca19.9 (>22 U/mL) - 66% Elevated CEA (>4.0 ng/mL) - 20%	**Focal or diffuse gallbladder wall thickening** ▪ USS - 63 ▪ CT - 36 ▪ MRI - 22 **Intramural hypoechoic nodules** ▪ CT - 15 ▪ MRI – 7 **Cholecystolithiasis** – 93 **Hepatic abscesses** - 6	▪ Laparoscopic cholecystectomy - 40/100 ▪ Open cholecystectomy - 60/100 **Additional procedures:** ▪ Radical cholecystectomy - 2 ▪ Hepatectomy - 41 ▪ Extrahepatic biliary tract exploration - 39 ▪ Choledochorrhaphy - 12 ▪ T Tube drainage – 22 ▪ Pancreaticoduodenectomy - 2	Histopathological diagnosis of XGC from the gallbladder specimens. Findings not provided in the study.	Mortality - 0 Morbidity: ▪ Wound infection - 3 ▪ Abdominal infection - 7 ▪ Pulmonary infection - 2 ▪ DVT - 1
Bolukbasi (2020)	Sample size: 34 ▪ M 17 ▪ F 17 Mean age: 53	**Fever** - 12 (35%) **Dyspepsia** - 12 (35%) **Positive Murphy sign** - 17 (50%) **Abdominal pain** - 29 (85%) **Vomiting** - 13 (38%) **Jaundice** - 6 (18%) **Weight loss** - 5 (15%) **Palpable mass** - 7 (21%) **Anorexia** - 12 (35%) **Charcot’s Triad** - 1 (3%)	*None provided*	**USS** - 34 (100%) ▪ GB stones - 28 (82.3%) ▪ Sludge - 10 (29%) ▪ Adenomyomatosis - 6 (17.6%) ▪ Polyp - 2 (6%) ▪ GB wall thickening - 21 (61.8%) **Focal or diffuse wall thickening** ▪ USS - 17 (50%) ▪ CT - 7 (58%) ▪ MRI - 1 (33.3%)	▪ Laparoscopic cholecystectomy - 25/34 ▪ Open total cholecystectomy - 6/34 ▪ Open radical cholecystectomy - 1/34 ▪ Open partial cholecystectomy - 1/34 ▪ Open cholecystectomy and primary suturing of common hepatic fistula over T tube and ERCP-sphincterotomy-stenting - 1/34	Foamy macrophages were the diagnostic feature in this study. Associated features were marked fibrosis, cholesterol clefts and multinucleated giant cells. Post cholecystectomy specimens: ▪ Diffuse XGC - 28 (82%) ▪ Focal XGC - 6 (17%)	Mortality - N/A Morbidity: ▪ Wound infection with intra-abdominal abscess- 2 (6%) ▪ Wound infection without abscess - 4 (12%)
Milkhu (2018)	Sample size: 14 ▪ M n/a ▪ F n/a Mean age: N/A	*None provided*	*None provided*	**USS/MRCP/CT** ▪ No abnormalities - 3/14 (21%) ▪ Oedematous wall/cholecystitis/inflammatory changes/free fluid in GB fossa/thick-walled gallbladder - 11 (79%) ▪ Irregular GB wall - 1 (7%) ▪ Empyema −1 (7%) ▪ Marked gallbladder wall thickening with low attenuation mural nodules - 14% (2)	Laparoscopic cholecystectomy - 100% (14)	Histopathological diagnosis of XGC from the gallbladder specimens. Findings not provided in the study. No signs of dysplasia or malignancy seen.	Mortality - N/A Morbidity: ▪ Perforation rate - 36% (5)
Alotaibi (2022)	Sample size: 10 ▪ M 6 ▪ F 4 Mean age: 47	Features of biliary colic - 5 (50%) Features of acute cholecystitis - 3 (30%) Features of biliary pancreatitis - 1 (10%) Features of obstructive jaundice - 1/10 (10%) Average symptom duration - 5.2 weeks (1–12)	Mean Values WBC - 8.3 × 10^3 (3.94–15.52) Total bilirubin - 0.71 mg/dl (0.18–1.81) INR - 0.9 (1.2–0.98)	**US** ▪ Multiple stones - 4 (40%) ▪ Biliary mud - 1 (10%) ▪ Mucocele - 1 (10%) ▪ Wall oedema - 1 (10%) ▪ Pericholecystic fluid - 2 (20%) ▪ Mural diverticula - 1 (10%) ▪ Pyocele - 1 (10%) ▪ Soft tissue mass (4 cm) inside gallbladder - 1 (10%) Average wall thickness - 5 mm (2–37) Average stone size - 7 mm (5–13) Average CBD diameter - 4.5 mm (2–7)	▪ Preoperative ERCP and Stenting - 2 (20%) ▪ Laparoscopic cholecystectomy - 9 (90%) ▪ Open cholecystectomy - 1 (10%) ▪ Drain insertion - 4 (40%) ▪ Liver bed excision for frozen section- 1 (10%)	Findings suggestive of XGC included: Ulcerated mucosa Foamy histiocyte and macrophages Multinucleated giant cells and granulomas Cholesterol granuloma Transmural extensive macrophages infiltrate	Mortality: n/a Morbidity: ▪ No postoperative complications over median follow up of 24 months (11–30)
Makimoto (2020)	Sample size: 31 ▪ M 20 ▪ F 11 Mean age: 71.7 (55–91)	**Abdominal pain** - 18 (58.1%) **Jaundice** - 5 (16.1%) **Fever** - 3 (9.7%) **Nausea and vomiting** - 2 (6.5%) **Abdominal distension** - 1 (3.2%) **No symptom** - 2 (6.5%)	CA19-9 raised in four patients. CEA raised in one patient.	**MRI:** intramural T2 high signal intensity - 2/4 **US**: wall thickening - 5. **CT**: ▪ focal wall thickening - 4 ▪ Diffuse wall thickening - 1 **MRI**; ▪ Focal wall thickening - 3 ▪ Diffuse wall thickening - 1 ▪ CBD Stenosis - 1 ▪ Intramural T2 High Signal Intensity - 2 **FDG-PET;** abnormal uptake - 4/5	▪ Laparoscopic cholecystectomy = 11 (35.4%) ▪ Laparoscopic cholecystectomy with conversion to open = 12 (38.7%) ▪ Open cholecystectomy = 4 (12.9%) ▪ Open cholecystectomy with gallbladder bed resection with a surgical margin = 2 (6.5%) ▪ Cholecystectomy, hepatic resection, extrahepatic bile duct resection with Roux-en-Y hepaticojejunostomy = 1 (3.2%) Due to Mirizzi Syndrome ▪ Pancreaticoduodenectomy = 1 (3.2%) Preoperative biliary drainage = 21 (67.7%) ▪ ERBD = 11 ▪ EST = 10 ▪ PTBD = 1	Diagnosis of XGC was based on the following histopathological findings: presence of diffuse or focal mural changes accompanied with xanthoma cells (i.e. foamy histiocytes containing lipid and bile pigment), multinucleated giant histiocytes, and acute or chronic inflammatory cells	Morbidity ▪ >1 L intraoperative blood loss = 1 ▪ Postoperative intra-abdominal abscess = 2 ▪ Postoperative wound infection = 1
Yucel (2016)	Sample size: 108 ▪ M 56 ▪ F 52 Mean age: 62.3 (27–93)	**Abdominal pain** - 108 (100%) **Chronic Cholecystitis** - 77 (71.3%) **Acute Cholecystitis** - 31 (28.7%) **Mirizzi’s Syndrome** - 15 (13.8%) **Choledocholithiasis** - 12 (11.1%) **Cholangitis** - 5 (4.6%) **Acute Pancreatitis** - 5 (4.6%)	None provided	**Focal or diffuse wall thickening** ▪ US 71 (65.7%) ▪ CT 13 (52%) ▪ MRI 16 (55.1%) **Adenomyomatosis** ▪ MRI 1 **Choledocholithiasis** ▪ MRCP 12/25 **Mirizzi Syndrome** ▪ MRCP 3	Procedural management ▪ ERCP with sphincterotomy + stone extraction successful in 10 patients. ▪ Five patients treated with percutaneous cholecystostomy for symptomatic relief Surgical management ▪ Laparoscopic cholecystectomy = 31 (28.7%) ▪ Laparoscopic cholecystectomy with conversion to Open = 23 (21.3%) ▪ Open cholecystectomy = 54 (50%) ▪ Partial cholecystectomy = 11 (10.2%)	Diffuse XGC confirmed in 91 patients (84.3%) and Focal involvement in 17 patients (15.7%) ▪ 98 patients had gallbladder calculi (90.7%) ▪ two patients had hydatid cysts ▪ one patient had concomitant gallbladder carcinoma. ▪ one patient with adenocarcinoma accompanying XGC. Intraoperative frozen section performed on 18 patients. 16 confirmed as XGC. Two confirmed as gallbladder carcinoma.	Average hospital stay 5.1 days (1–32) Complications in 10 patients (9.25%) ▪ 9 Wound infections ▪ 1 DVT No mortality
Saritas (2020)	Sample size: 79 ▪ M 52 ▪ F 27 Mean age: 65.8 (36–97)	**Abdominal Pain** - 50 **Abdominal pain + Nausea** - 22 **Abdominal pain + Fever** - 15 **Jaundice** - 7	Median Values CA 19-9 40.3 U/mL. CEA 1.6 ng/mL.	**US (79/79):** ▪ Cholelithiasis 70. ▪ Intramural thickening 47 (59.5%). ▪ Malignancy Suspicion 6 **CT (38/79):** ▪ Cholelithiasis 34. ▪ Intramural thickening 31 (81.6%). ▪ Malignancy Suspicion 15 **MRI (22/79)** ▪ Cholelithiasis 18. ▪ Intramural thickening 18 (81.8%). ▪ Malignancy Suspicion 8	▪ Laparoscopic cholecystectomy = 24 (30.4%) ▪ Laparoscopic cholecystectomy with conversion to Open = 3 (3.8%) ▪ Open cholecystectomy = 48 (60.8%) ▪ Open cholecystectomy with, other organ resection = 4 (5.1%) ▪ Pancreaticoduodenectomy for tumour of ampulla of vater = 1 ▪ Right hepatectomy for Klatskin tumour = 1 ▪ Roux-en-Y Hepaticojejunostomy for cholangiocellular carcinoma = 2	79 patients diagnosed with XGC. Criteria for XGC in the pathology specimens were the presence of histiocytes, cholesterol deposits, lipids, and focal or widespread wall enlargement. Two patients with accompanying cholangiocellular carcinoma. One patient with tumour of ampulla of vater One patient with klatskin tumour	Duodenal injury - 1 Common Bile Duct Injury - 1 Wound infection - 4 Bile fistula - 2 Pulmonary complication - 4 Length of hospital stay 4.6 ± 3.6
Khan (2021)	Sample size 8 ▪ M 5 ▪ F 3 Age range 24–69 All eight patients suspected of GBCa	**Chronic Cholecystitis** ▪ RUQ pain (3) ▪ Abdominal distension (2) ▪ Anorexia (4) **Acute Cholecystitis** ▪ RUQ pain (3) ▪ High grade fever (>38) (3) ▪ Elevated WBC (2) ▪ Nausea vomiting (3) **Jaundice** - 2 **RUQ mass** − 3	Increased CA19-9 (>22 U/mL) - 6 Increased CEA (>4.0 ng/mL) - 2	**USS (8);** thickened gallbladder wall - 6 **CT (5):** ▪ Thickened gallbladder wall - 5. ▪ diffuse thickening - 4 ▪ intramural hyperattenuated nodules - 3 ▪ Blurred liver/gallbladder margins - 5 ▪ hepatic infiltration - 4 ▪ infiltration of omentum, colon, duodenum, or stomach - 3 ▪ Regional Lymph Node enlargement - 3	▪ Open Radical Surgery (5) ▪ Laparoscopic cholecystectomy with conversion to Open (3)	All cases were diagnostic of XGC with the following characteristics: ▪ All cases showed thickened GB Wall ▪ a mass lesion in three cases ▪ seven cases transmural inflammation comprising sheets of foamy histiocytes and dense inflammatory infiltrate ▪ five cases with multinucleated giant cell ▪ three cases with cholesterol clefts ▪ Lymph nodes of seven patients showed reactive change. One LN with coalescent epithelioid granuloma suggestive of Tuberculosis	No further details provided
Domínguez-Comesaña (2019)	Sample size 63 ▪ M 36 ▪ F 27 Mean age 65.8	Preoperative diagnosis: **Acute Cholecystitis** – 28 **Biliary Colic** – 22 **Choledocholithiasis** – 3 **Neoplasia** – 3 **Biliary Fistula** – 3 **Acute Pancreatitis** - 3 **Cholangitis** - 1	Blood results not provided	**US (63/63)** Diffuse gallbladder wall thickening - 35 (55.5%) **CT (12/63)** Diffuse gallbladder wall thickening - 7 (58.3%)	▪ Laparoscopic cholecystectomy = 27 ▪ Laparoscopic cholecystectomy converted to open cholecystectomy = 18 ▪ Open cholecystectomy = 18 Cause of conversion: ▪ Difficult dissection *n* = 14 ▪ Dense adhesions *n* = 2 ▪ Haemorrhage *n* = 2	All 63 patients confirmed to have XGC after surgery was based on the following criteria: presence of foamy histiocytes containing lipids and bile pigments, giant multinucleated histiocytes and inflam- matory cells Intraoperative Frozen section negative for malignancy *n* = 3, for patients suspected of XGC.	Mortality = 2 Death (Both over 85 years old) Morbidity - 15 ▪ Incisional surgical site infection - 4 ▪ Intra-abdominal abscess - 3 ▪ Intestinal perforation - 1 ▪ Hepatic abscess - 1 ▪ Bile duct injury - 2 ▪ Intra-abdominal haemorrhage - 1 ▪ Wound/abdominal wall hematoma - 2 ▪ Evisceration - 1 ▪ Pneumonia - 1 Average Length of Stay ▪ 7.19 ± 6.6 days (range: 1–35 days)
Kamtam (2020)	Sample size 60 ▪ M 26 ▪ F 34 Mean age 50.6 (15–76)	**RUQ Pain** 63.6% **Epigastric Pain** - 11 (18.3%) **Fever** - 22 (36.6%) **Vomiting** − 16 (27.7%) **Jaundice** − 7 (11.7%) **PMHx of ERCP treated choledocholithiasis** - 9 **Fever + Severe RUQ Pain** - 1	Mean values WBC 7.6 (SD3.6) × 109/L Bilirubin 0.7 (SD0.3) mg/dL AST 45.1 (SD34.2) IU/L ALT 37 (SD24.8) IU/L ALP 198.2 (SD78.8) U/L	**US:** ▪ Calculi in gallbladder - 55 (91.7%) ▪ Diffusely thickened gallbladder wall >4 mm - 58 (96.7%) ▪ Dilated CBD >8 mm - 16 (26.7%) ▪ 9 with Gallstone in CBD ▪ Could not be ruled out for carcinoma - 11 (18.3%) **CT (11)** ▪ Irregularly enhanced thickening of gallbladder wall - 11 (100%) ▪ Calculi - 9 (81.8%) ▪ Pericholecystic collection - 3 (27.3%) ▪ Malignant imaging - 3 (27.3%)	Preoperative ERCP 9 out of 16 patients with dilated CBD underwent ERCP.	Diagnosed by histopathological examination of cholecystectomy specimens. Findings diagnostic of XGC included: ▪ focal or diffuse mural changes ▪ foamy histiocytes containing lipids or bile pigments ▪ giant multinucleated histiocytes, ▪ acute or chronic inflammatory cells	

The common clinical features of this disorder include right upper quadrant pain, nausea, vomiting and weight loss. In review of the combined data from all case reports and case series, 85% of cases reported abdominal pain as a presenting symptom. Other commonly reported features were fever (19%), jaundice (17%) and weight loss (22%). Significantly, almost two-thirds (60%) of episodes reported at least 1 month of symptoms prior to presentation. Physical examination may reveal a positive murphy’s sign, jaundice and a right upper quadrant mass [12].

Serological evaluation remains non-specific; and therefore, may not benefit the diagnosis of XGC. Liver function derangement or inflammatory marker elevation (white cell count or C-reactive protein) may correspond with episodes of XGC. For example, derangement of Liver Function Tests (LFTs) outside of normal laboratory values was seen in 35% (41/118) of cases, and raised white cell count in 31% (40/127) of cases identified through our literature review. However, these findings remain non-specific as similar derangements may be reflected in other common hepato-biliary conditions such as cholecystitis or choledocholithiasis and cannot predict with certainty the diagnosis of XGC.

Furthermore, tumour markers such as Ca 19.9 correlate well with the incidence of cholangiocarcinoma, but in isolated XGC cases may be elevated so do not provide diagnostic certainty [13, 14]. Indeed, as seen through our literature review, a total of 111 cases were identified, which recorded a value for the tumour marker Ca 19-9, and in almost two-thirds of cases (63%), this value was elevated.

Imaging remains a cornerstone of surgical evaluation. The locally invasive nature of the disease seen in radiological assessment can easily be mistaken for cholangiocarcinoma. The most characteristic ultrasonographic feature of XGC is the presence of hypoechoic lesions or nodules, which may be present in 35% of cases [15]. Our analysis found a similar prevalence with 18% (16/89) of cases demonstrating intramural nodules or bands. A further diagnostic characteristic of XGC is gallbladder wall thickening. In a review of 26 patients Parra *et al*. [15] observed that the wall thickening was hyperechoic comparted to liver in 100% of cases of XGC, with the presence of the characteristic finding of hypoechoic nodules observed in 15% of cases.

CT commonly shows continuous mucosal line enhancement, widespread submucosal hypo-attenuated nodules or bands, and often diffuse rather than focal wall thickening, where focal wall thickening is more likely to be observed in cholangiocarcinoma [16]. An analysis by Rammohan [17] of 76 patients with a pre-operative diagnosis of cholangiocarcinoma found that of the 16 cases later found to represent XGC, almost 70% (11/16) had one of these three radiological findings.

Through retrospective analysis of 22 cases, Goshima [18] found that where three of the following five features were present in combination this correlated with a sensitivity of 83%, a specificity of 100% and an accuracy of 91% for XGC. These five features included: diffuse gallbladder wall thickening, a continuous mucosal line, intramural hypo-attenuated nodules, the absence of macroscopic hepatic invasion and the absence of intra-hepatic bile duct dilatation. The continuity of the hyper-enhancing mucosal line is a feature of the underlying pathophysiology of the disease that effects the gallbladder wall, and therefore the mucosal surface is intact; in contrary to cholangiocarcinoma where the disease arises from the gallbladder epithelium leading to mucosal disruption. Indeed, mucosal line disruption has been observed in 82.2% of cases of cholangiocarcinoma [19].

Pre-operative differentiation of XGC from other inflammatory or malignant pathologies based on clinical presentation, serological evaluation and radiological findings is difficult. As such, XGC is a histological diagnosis. Macroscopic features commonly include gallbladder wall thickening fibrous adhesions, fistulous tracts and ceroid (wax-like) yellowed nodules or streaks. Many of these features share characteristics with malignant processes, which make diagnosis based on macroscopic assessment problematic [20]. Diagnostic features on microscopic evaluation are xanthogranulomateous infiltration, lipid-laden macrophages and the presence of fibroblasts, in the absence of dysplastic cells.

The definitive treatment for XGC is cholecystectomy. Laparoscopic cholecystectomy may be attempted, but conversion to open cholecystectomy is commonly required. In our analysis of 629 surgical cases of XGC, 11% (67/629) were converted from laparoscopic cases. Depending on the chronicity and severity of the disease progression, there may be dense fibrosis, fistulas and extensive inflammation present making laparotomy an often preferable approach. Indeed, over half (52%) of all cases identified required an open operation.

## CONCLUSION

XGC is a rare disorder associated with submucosal abscesses secondary to extravasated bile into the gallbladder wall. The diagnosis of XGC may be considered more likely in the presence of acute cholecystitis, choledocholithiasis and cholelithiasis than without these comorbidities. While radiological features such as large areas of submucosal hypo-attenuated nodules, a continuous mucosal line in a thickened gallbladder wall and the presence of gallstones are more suggestive of XGC. Differentiating XGC from cholangiocarcinoma is difficult based upon clinical and radiological parameters, with definitive diagnosis reliant upon histological assessment. However, an increased accuracy of preoperative diagnosis and creation of a diagnostic aid could help avoid extended invasive resections.

## Data Availability

The data are deemed confidential and under ethics cannot be disseminated openly due to confidentiality and privacy.
